# Mortality and its association with CD4 cell count and hemoglobin level among children on antiretroviral therapy in Ethiopia: a systematic review and meta-analysis

**DOI:** 10.1186/s41182-020-00267-y

**Published:** 2020-09-21

**Authors:** Chalachew Adugna Wubneh, Getaneh Mulualem Belay

**Affiliations:** Department of Pediatrics and Child Health Nursing, School of Nursing, College of Medicine and Health Sciences, University of Gondar, Gondar, Ethiopia

**Keywords:** ART, CD4, Children, Ethiopia, Hemoglobin, Meta-analysis, Mortality, Systematic review

## Abstract

**Background:**

Even though there are advancements in HIV/AIDS prevention and treatment approach, HIV continues to be a global challenge. Pediatrics HIV is one of the challenges in the reduction of child mortality particularly in less developed countries like Ethiopia. Therefore, this study aims to estimate the pooled proportion of child mortality and the effect of hemoglobin level and CD4 cell count among children on antiretroviral therapy in Ethiopia.

**Method:**

All published were articles searched using PubMed, EMBASE, Google Scholar, and Web of Science database. Besides, Ethiopian institutional research repositories and reference lists of included studies were used*.* We limited the searching to studies conducted in Ethiopia and written in the English language. Studies that were done in a cohort, cross-sectional, and case-control study design were considered for the review. The weighted inverse variance random effects model was applied, and the overall variations between studies were checked by using heterogeneity test Higgins’s (*I*^2^). Subgroup analysis by region and year of publication was conducted**.** All of the included articles were assessed using the Joanna Briggs Institute (JBI) quality appraisal criteria. In addition, publication bias was also checked with Egger’s regression test and the funnel plot. Based on the results, trim and fill analysis was performed to manage the publication bias.

**Result:**

A total of 16 studies with 7047 participants were included in this systematic review and meta-analysis. The overall pooled proportion of mortality among children on antiretroviral therapy (ART) was found to be 11.78% (95% CI 9.34, 14.23). In subgroup analysis, the highest child mortality was observed in the Amhara region 16.76 % (95% CI 9.63, 23.90) and the lowest is in the Tigray region 4.81% (95% CI 2.75, 6.87). Besides, the proportion of mortality among children with low CD4 count and hemoglobin level was 2.42 (AOR = 2.42, 95% CI 1.65, 3.56) and 3.24 (AOR = 3.24, 95% CI 1.51, 6.93) times higher compared to their counterparts, respectively.

**Conclusion:**

The proportion of mortality among children on ART was high in Ethiopia. Those children who had low CD4 cell count and low hemoglobin levels at baseline need special attention, treatment, and care.

**Trial registration:**

The protocol of this systematic review and meta-analysis has been registered in PROSPERO with the registration number CRD42018113077.

## Background

Human immunodeficiency virus (HIV) is one of the global pandemic infectious diseases that cause major survival challenges of humankind. Globally, 37.9 million people are living with HIV by the end of 2018; of these 20.6 million lives in eastern and South Africa, 1.7 million people acquired new HIV infection and 160,000 were infants [1]. Worldwide in 2018, 770,000 peoples died from HIV-related causes [2]. One of the major challenges in child morbidity and mortality reduction is pediatric HIV [3]. According to the World Health Organization (WHO) report in 2019, 89% of low- and middle-income countries, including Ethiopia, has adopted treat all strategies for children from the international policy [4, 5]. As a result, the introduction of this highly active antiretroviral therapy (HAART) significantly reduced HIV-related mortality and morbidity among children infected with HIV [6–10]. According to global estimation, 1.7 million children under the age of 15 years were living with HIV and 100,000 children died with acquired immune deficiency syndrome (AIDS)-related causes [11]. Although significant progress has been made in the accessibility of ART and improved program implementation, child mortality even after ART initiation is a challenge for Africa including Ethiopia [12–14]. Different observational studies have been conducted so far in different parts of Ethiopia with variation in time and place. Those studies have reported the magnitude and predictors of mortality among children taking ART. But there is inconsistency in the magnitude and predictors of mortality. Especially, the effect of baseline hemoglobin and low CD4 cell count on child mortality after the initiation of ART is an important issue to be studied. These predictors were reported as statistically significant in some studies, whereas in other studies, they were not significant. Besides, in sub-Saharan Africa (SSA), including Ethiopia, one systemic review and meta-analysis had been conducted on the proportion of child mortality [14], but there was no national study which assesses on the proportion of child mortality and the effect of baseline hemoglobin and CD4 cell count on the child mortality. Therefore, this systemic review and meta-analysis aims to estimate the national magnitude of child mortality after ART initiation and its association with baseline hemoglobin and CD4 cell count in Ethiopia.

## Method

### Reporting

To report the findings of this systematic review and meta-analysis, the Preferred Reporting Items for Systematic Review and Meta-analysis (PRISMA) guideline was used [15] (Additional file 1). The protocol of this study was registered in PROSPERO with a registration number of CRD42018113077.

### Databases and search strategy

An advanced search was carried out in electronic databases including, PubMed, EMBASE, Web of Science, and Google Scholar. In addition, Ethiopian university research repository, gray literature from Google, and reference lists of included studies were searched. Moreover, we searched studies manually. We had been searching for articles until April 15, 2020. The search focused on the studies that reported the proportion of mortality among children on ART and/or its association with at least hemoglobin and/or CD4 count. The searching was conducted using the following terms and/or phrases: “Mortality,” “Death,” “Treatment outcome,” “Survival,” “Attrition,” “HIV,” “Human Immune Deficiency Virus,” “ Acquired Immune Deficiency Syndrome,” “ART,” “Antiretroviral Therapy,” “Highly Active Antiretroviral Therapy,” “Prevalence,” “Proportion,” “Associated Factors,” “Predictors,” “Hemoglobin,” “Hgb,” “CD4 Count,” “Children,” “Child,” “Pediatrics,” “Paediatrics,” “Infant,” “ Neonates,” and “ Ethiopia.” The searching strings were developed by using “AND” and “OR” Boolean operators (Additional file 2).

### Inclusion and exclusion criteria

We have included the studies if they fulfill the following inclusion criteria: (1) studies conducted in Ethiopia (2); studies conducted on children < 15 years of age (3); observational studies, including cross-sectional, cohort, and case-control studies (4); studies that reported proportion of mortality and/or at least its association with hemoglobin and/or CD4 count (5); the outcome was the proportion of mortality among children on ART; and (6) studies published in the English language. On the other hand, qualitative studies, trials, and citations without full-text were excluded.

### Quality assessment and study selection

To remove duplicated studies, the Endnote version 7 (Thomson Reuters, London) reference manager was used. Two reviewers (CAW and GMB) independently screened the titles and abstracts to consider the articles in the full-text review. Two investigators (CAW and GMB) assessed the quality of the studies using Joanna Briggs Institute quality appraisal criteria (JBI) [15]. The JBI critical appraisal checklist for cohort studies was employed (Additional file 3). The discrepancy was solved by consensus. Studies that got 50% and above of the quality scale were considered low risk and included in the meta-analysis.

### Data extraction

Two independent investigators (CAW and GMB) extracted the data. Any disagreement was solved by repeating the procedure. Information about the first author and year of publication, study region, setting, design, population, sample size, follow-up period, the proportion of mortality, and the odds ratio of hemoglobin and CD4 count were extracted.

### Summary measures

Child mortality in this systematic review and meta-analysis was considered as the death of confirmed HIV-positive children after ART started as described in the primary articles. Anemia at baseline was defined as low hemoglobin level less than 10 g/dl. Low CD4 cell count (below the threshold) at baseline was defined in different age categories as it varies in the different age groups of children. Therefore, it was defined as (for up to 12 months CD4 < 1500/mm^3^, 12–35 months < 750/mm^3^, 36–59 months < 350/mm^3^, and ≥ 5 years < 200/mm^3^) as it was defined from primary studies included in the analysis [16–23].

### Data analysis

A weighted inverse variance random effects model [24] was used to estimate the proportion of mortality among children on ART and the odds ratio of hemoglobin and CD4 count. The variation in the pooled estimates of the proportion of mortality among children on ART was adjusted through subgroup analysis by the region where the study conducted and time after and before the implementation of test and treat strategies of ART treatment. Heterogeneity among included studies was checked using *I*^2^ statistics where 25, 50, and 75% represent low, moderate, and high heterogeneity, respectively [25]. To assess the presence of publication bias, funnel plot and Egger’s regression test were used. Trim and fill analysis were conducted to manage the publication bias. Moreover, a sensitivity analysis was conveyed to check the stability of the summary estimate. We used STATA version 11 statistical software to conduct this meta-analysis.

## Result

### Literature search results

**Fig. 1:**
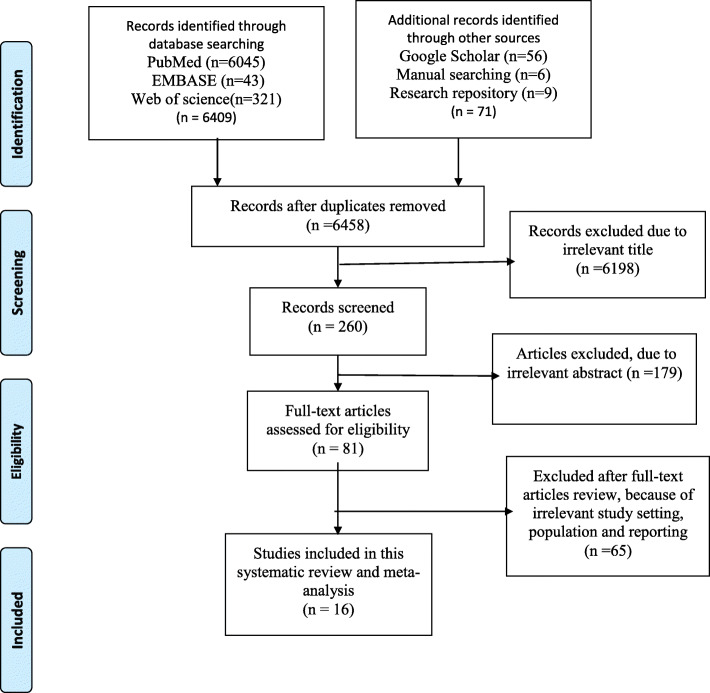
Flow diagram of articles selection and screening process

We found a total of 6480 articles from different electronic databases (PubMed, EMBASE, Google Scholar, and Web of Science) and manual searching from Ethiopian university repository and reference lists of screened studies of which 22 were removed because of duplicates. By reviewing the titles and abstracts, 6198 and 179 were excluded from analysis, respectively. Finally, 16 articles were considered as fitted for the analysis from 81 studies selected for full-text review. The details of selection process articles are described in (Fig. 1).

### Characteristics of included studies

On the whole, 16 studies with 7047 study participants had been included in the study. Except for one study which is a prospective follow cohort study [26], the remaining 15 studies were retrospectively followed up [16–23, 27–33]. All of the studies had been conducted from 2005 to 2017 in different follow-up periods with a maximum of 10 years and a minim of 1 year. Articles published between 2009 and 2019 were included in this review (Table 1).

**Table 1:** Characteristics of included studies that reported the proportion of child mortality (*n* = 16)

Author	Year of publication	Region	Follow-up period	Study design	Study population	Sample size	Number of outcome	Percent (%)
Koye et al.	2012	Amhara	2006–2011	Retrospective cohort	< 15 year	549	41	7.50
Gebremedhin et al.	2013	Tigray	2006–2011	Retrospective cohort	< 15 year	416	20	4.81
Taye et al.	2010	Addis Ababa	2005–2008	Retrospective cohort	< 15 year	475	42	8.80
Asfawesen et al.	2011	Addis Ababa	2005–2008	Retrospective cohort	< 15 year	482	13	7.50
Dube et al.	2017	Addis Ababa	2008–2010	Retrospective cohort	< 15 year	757	51	6.70
Ebissa et al.	2015	Addis Ababa	2008–2009	Retrospective cohort	1–12 year	556	58	10.40
Sidamo et al.	2017	SNNPRS	2009–2016	Retrospective cohort	≤ 14 year	421	65	15.40
Biru et al.	2018	Addis Ababa	2014–2016	Prospective cohort	< 14 year	304	6	25.00
Atnafu and Wencheko	2012	Amhara	2007–2009	Retrospective cohort	< 15 year	255	71	27.84
Netsanet et al	2009	Oromia	2005–2008	Retrospective cohort	< 14 year	96	7	7.30
Andargie et al.	2018	Amhara	2008–2013	Retrospective cohort	< 15 year	269	46	17.10
Edessa	2015	Harari	2010–2013	Retrospective cohort	< 15 year	305	28	9.20
Kedir	2014	Oromia	2006–2010	Retrospective cohort	< 14 year	560	43	7.60
Mokgatle et al.	2016	Addis Ababa	2005–2012	Retrospective cohort	< 15 year	786	62	7.90
Alebel et al.	2018	Amhara	2012–2017	Retrospective cohort	< 15 year	390	38	9.70
Arage et al.	2019	Amhara	2005–2015	Retrospective cohort	< 15 year	426	97	22.90

### Quality of included studies

We found that all of the 16 studies included in this analysis were followed up; for this reason, the quality was assessed with the JBI quality appraisal checklist for follow-up studies, and studies that scored above 50% were considered as low risk and none of them were excluded after assessing the quality of studies. The result of the quality assessment ranges from 62.5 to 75% (Additional file 3).

### Meta-analysis

**Fig. 2:**
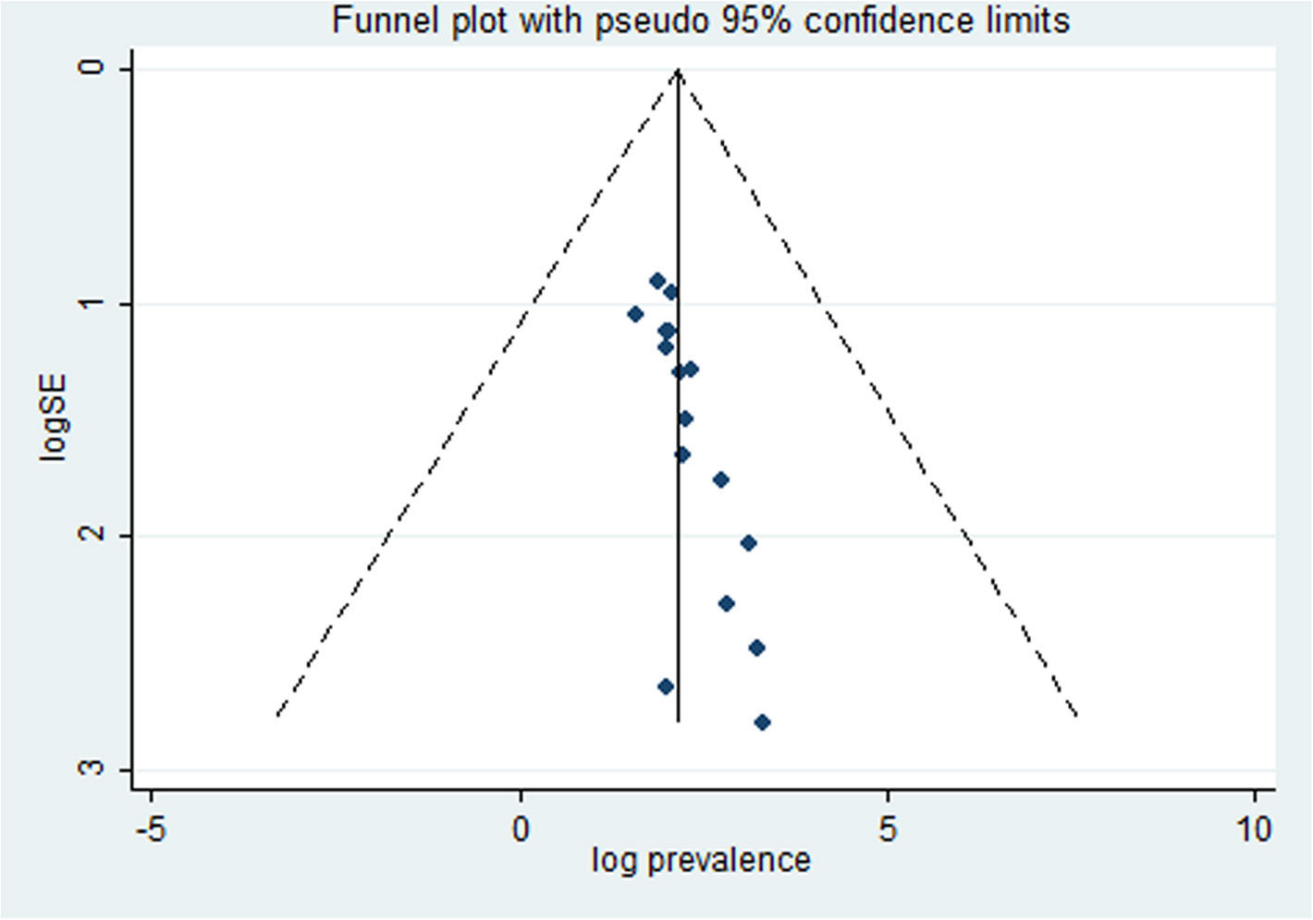
Funnel plot for publication bias. Log prevalence and logSE represented the log of the prevalence in the *x*-axis and log of the standard error in the *y*-axis, respectively

To assess the presence of publication bias, the funnel plot and Egger’s regression test were performed. Egger’s regression test was statically significant (0.000) which revealed the presence of publication bias. To handle this bias, trim and fill analysis was performed. Consequently, two articles have been added in the fill stage (Fig. 2).

### Mortality

**Fig. 3:**
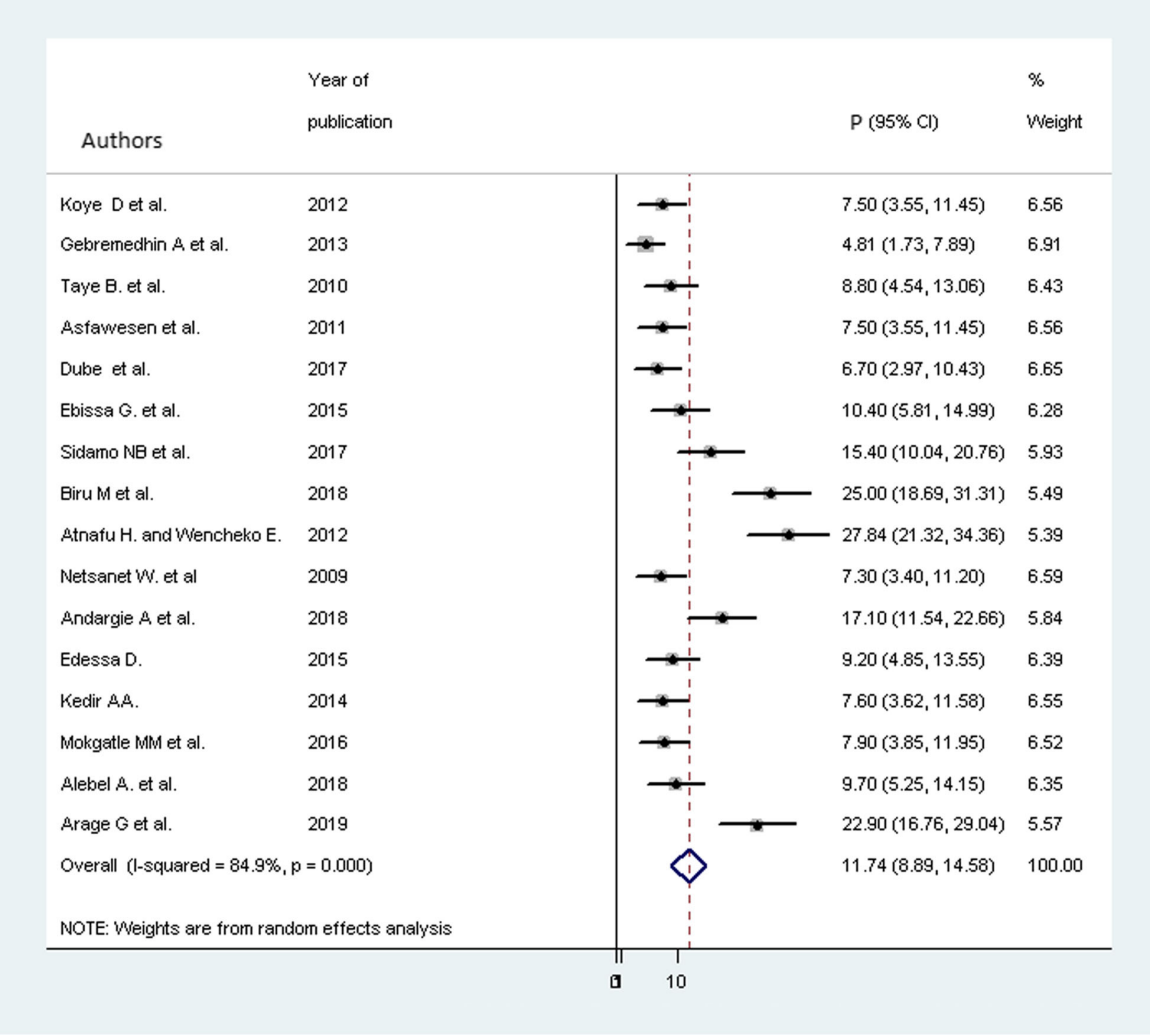
Forest plot for the proportion of child mortality with 95% CI. The midpoint and the length of each segment revealed the proportion and 95% CI of each study, respectively. The diamond shape indicated the pooled proportion

The overall pooled proportion of mortality after a trim and fill analysis among children on ART was 9.785% (95% CI 7.029, 2.542). Before meta-trim and fill analysis was performed, the pooled proportion of child mortality was 11.78 % (95% CI 9.34, 14.23). However, due to the presence of statically significant publication bias, meta-trim and fill analysis was conducted. Hence, two articles were filled and the proportion of mortality became 9.785% (95% CI 7.029, 2.542) (Fig. 3).

### Subgroup analysis

**Fig. 4:**
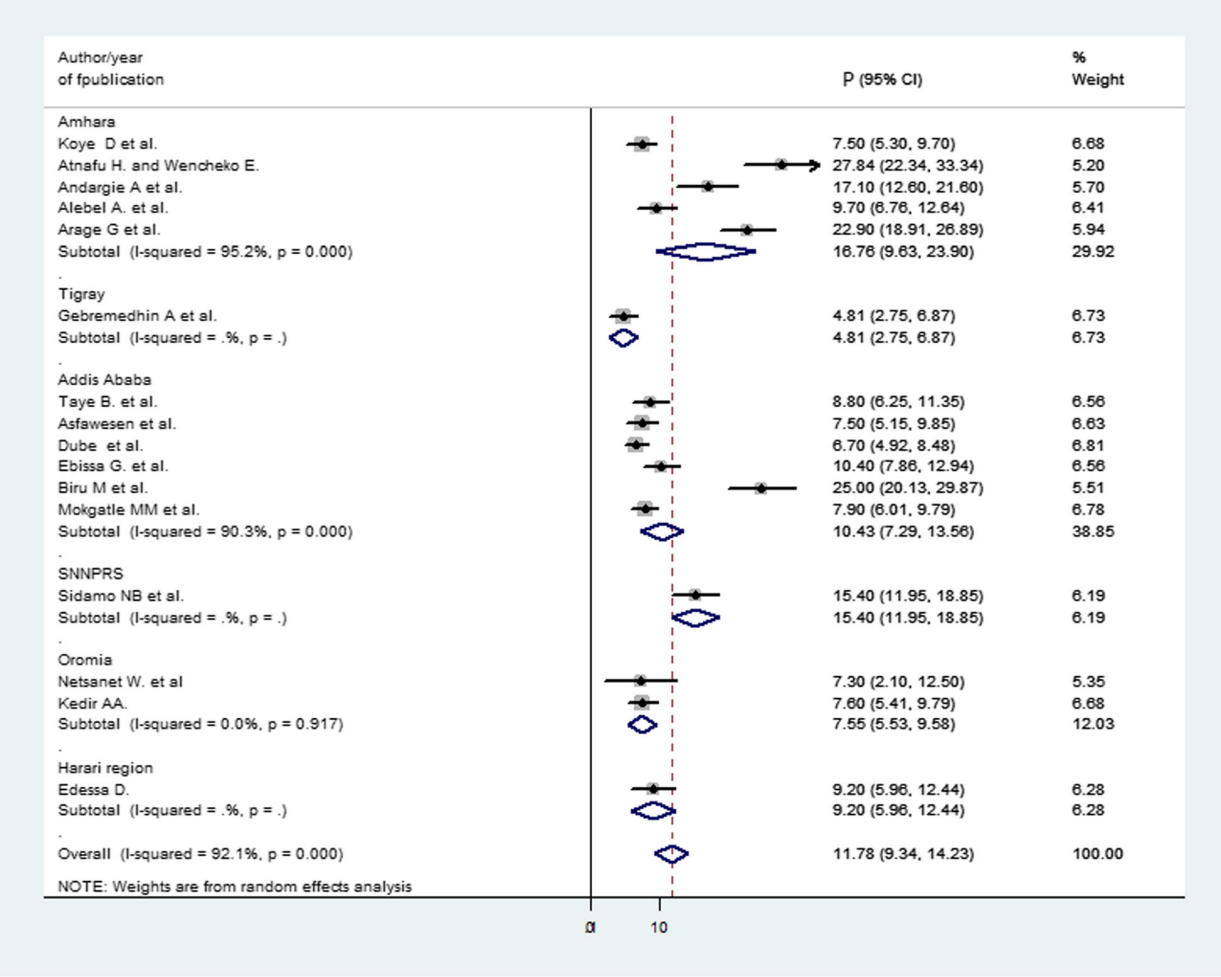
Forest plot for subgroup analysis of the proportion of child mortality by region. The midpoint and the length of each segment showed the proportion and 95% CI of each study, respectively. The diamond shape revealed the pooled proportion of child mortality in each region

Subgroup analysis was conducted by using the region and year of the primary studies published. From all studies which reported proportion of child mortality after ART initiation, 6 were from Addis, Ababa [18, 26–29, 33], 5 from Amhara [16, 20–23], 2 from Oromia [30, 32], 1 from Tigray [17], 1 from SNNPRS [19], and 1 from Harari [31]. In the subgroup analysis, the highest pooled proportion of mortality was reported in the Amhara region 16.76% (95% CI 9.63, 23.90) and the lowest in the Tigray 4.81% (95% CI 2.75, 6.87); subsequently, in Addis Ababa 10.43% (95% CI 7.29, 13.56), in Oromia 7.55% ( 95% CI 5.53, 9.58), in SNNPRS 15.40% (95% CI 11.95, 18.85), and in Harari 9.20% (95% CI 5.96, 12.44) were found (Fig. 4).

**Fig. 5:**
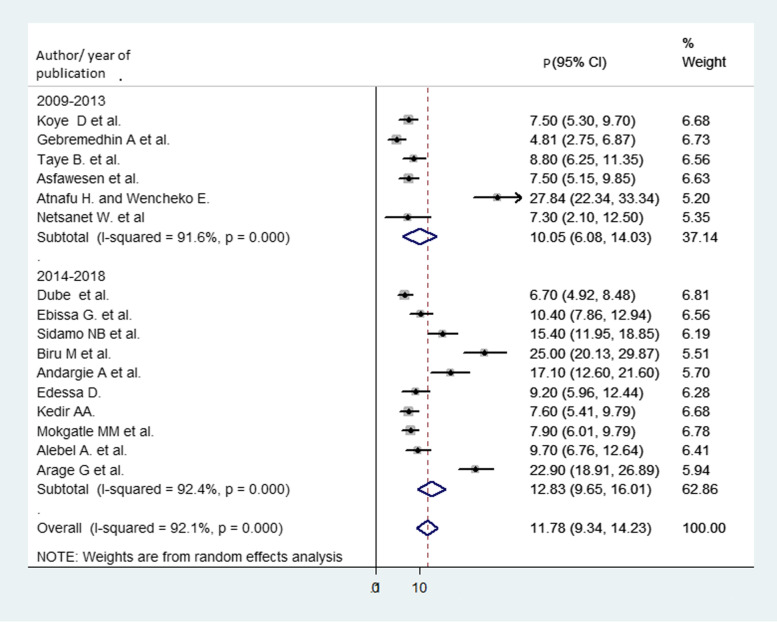
Forest plot for subgroup analysis of the proportion of child mortality by year of publication. The midpoint and the length of each segment revealed the proportion and 95% CI of each study, respectively. The diamond shape revealed the pooled proportion of child mortality

On the other hand, subgroup analysis was conducted by year of publication and categorized into two divisions, in which articles published between 2009 and 2013 were in one group and 2014 and 2019 in the other group. From this analysis, the proportion of mortality during articles published from 2009 to 2013 and 2014 to 2019 was 10.05% (95% CI 6.08, 14.03) and 12.83% (95% CI 9.65, 16.01), respectively (Fig. 5).

### The association between child mortality and baseline CD4 cell count

**Fig. 6:**
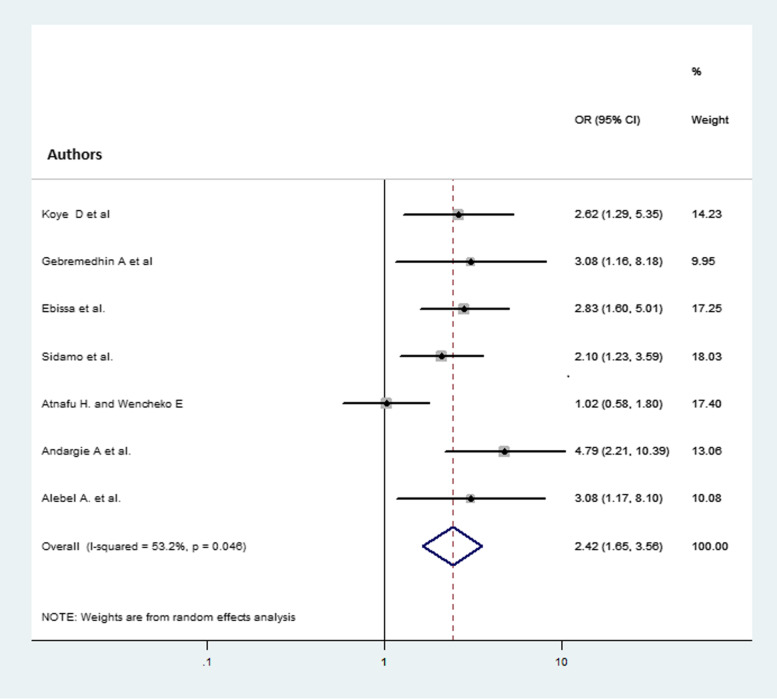
Forest plot that shows the association of child mortality and CD4 cell count at baseline. The midpoint and the length of each segment indicated the odds ratio and 95% CI of each study, respectively. The diamond shape showed the pooled odds ratio

From the total 16 studies included in the analysis of the pooled proportion of child mortality, 7 studies [16–22] for baseline CD4 cell count were eligible for analysis. Those children who had CD4 cell count below the threshold were 2.42 (AOR = 2.42, 95% CI 1.65, 3.56) times more likely to die compared with children having CD4 cell count above the threshold (Fig. 6).

### The association between child mortality and baseline hemoglobin level

**Fig. 7:**
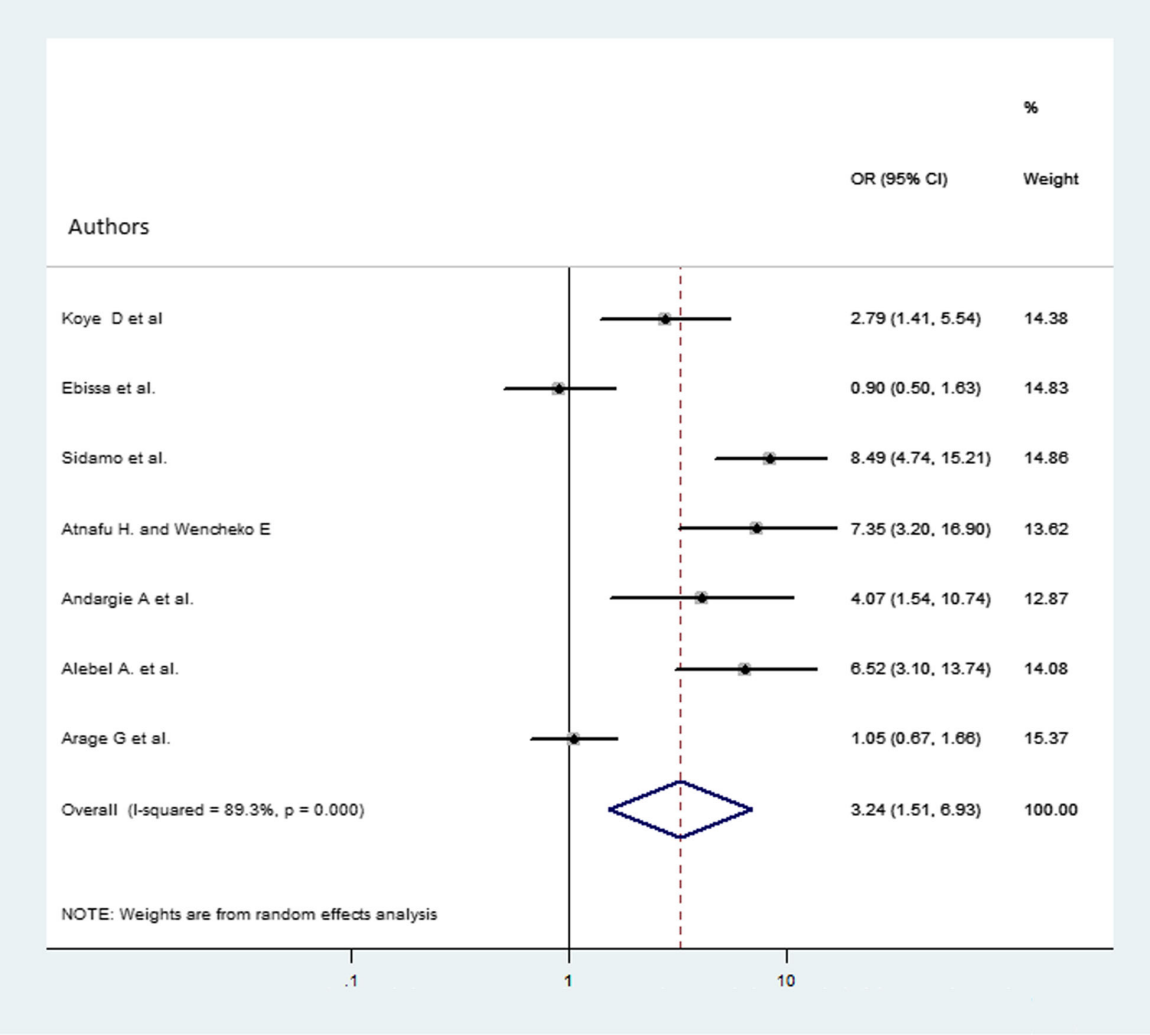
Forest plot that shows the association of child mortality and hemoglobin at baseline. The midpoint and the length of each segment indicated the odds ratio and 95% CI, respectively. The diamond shape showed the pooled odds ratio

A total of 7 studies [16, 18–23] were used in the analysis. The overall pooled odds ratio of mortality among children who had hemoglobin level less than 10 g/dl were 3.24 (AOR = 3.24, 95% CI 1.51, 6.93) more likely to die compared with children whose hemoglobin level was 10 g/dl and above (Fig. 7).

## Discussion

Child mortality is one of the global topics and is an indicator of the countries’ development. Even though there is good progress in the reduction of child mortality, still a large number of children die from this preventable disease [3]. Infectious diseases including HIV takes the major share for child mortality in Ethiopia. Therefore, this systematic review and meta-analysis is aimed to estimate child mortality due to HIV and contributing factors after the initiation of ART in Ethiopia.

This study revealed that 11.78% (95% CI 9.34, 14.23) of children died due to causes related to HIV infection after starting ART in Ethiopia. Nearly one of every twelve children is dead due to HIV infection and other related complications in Ethiopia. The result is slightly higher than the previous study conducted in SSA (7.9%) [14]. On the contrary, the finding of this study was lower than a systematic review conducted in low- and middle-income countries on the retention of pediatric patients on HIV treatment. The review reported that (37%) pediatric patients that were not in care are known to be dead [34]. This discrepancy might be because of the difference in the study periods. In addition, in the current systemic review and meta-analysis, most included articles was conducted in recent years after different interventions had been implemented to reduce mortality like test and treat and treat all strategies. These interventions have shown a significant reduction in mortality of children [4, 35, 36].

As a result of significant heterogeneity among studies, subgroup analysis was conducted by considering regional variation. As a result, the highest proportion of mortality was reported in the Amhara region (16.76%) followed by the SNNPRS region (15.4%), and the lowest proportion was observed in the Tigray region (4.81%). In addition to the proportion of death, this study also showed the impact of low hemoglobin level and CD4 cell count at baseline on mortality of children taking ART in Ethiopia. The findings indicated that those children who had low hemoglobin levels and CD4 cell count below the threshold at baseline were more likely to die compared with children who had a normal value.

Those children who had low hemoglobin levels were three times more likely to die as compared with children who had 10 g/dl and above hemoglobin level at baseline (AOR = 3.24, 95% CI 1.51, 6.93). Those children having low hemoglobin levels below 10 g/dl are anemic at baseline. Anemia is one of the comorbidities that cause a significant number to die especially in children [37]. The presence of anemia is one of the indicators for underlying undiagnosed and untreated comorbidity in children which need early diagnosis and treatment as equivalent to HIV treatment. On the other hand, some ART drugs can cause anemia directly. Especially, for those children having anemia at baseline, the condition will be aggravated with the drug side effects [38–41]. Besides this, the presence of anemia may indicate underlying nutritional problems which is the other major cause of child mortality in developing countries [42–44].

The other predictor of mortality was low CD4 cell count below the threshold at baseline. Those children who had low CD4 cell count at baseline was 2.4 times more likely to die compared with those who had CD4 cell count above the threshold (AOR = 2.42, 95% CI 1.65, 3.56). Those children having low CD4 cell count at baseline are exposed to a different infection that leads to different complications and finally leads to death [45, 46]. In Ethiopia, infectious diseases are the leading cause of death in children [12]. Infectious diseases are more severe and fatal in immunocompromised children than non-HIV-infected children [10]. As a result of low CD4 cell count, even the normal flora of the body can cause serious infections and complications that lead to death in children infected with HIV. The ART drug cannot treat the already developed opportunistic infections unless the underlying infections are treated.

### Strength and limitation

This study tried to show the national proportion of child mortality among children on ART in Ethiopia. Besides, this study showed that children who had low hemoglobin levels and CD4 cell count were more at risk for death compared with their counterparts. The study also has a limitation, and one of the limitations is it does not include representative papers in all regions of the country. In addition, we could not analyze more factors due to inconsistent measurements of factors and different operational definitions of variables.

## Conclusion

In Ethiopia, the mortality of children was high after ART initiation. Moreover, the proportion of mortality is higher among children having low CD4 cell count below the threshold and hemoglobin level less than 10 g/dl at baseline. Therefore, those children who had low CD4 cell count and low hemoglobin levels at baseline need special attention, treatment, and care.

## Supplementary information

**Additional file 1.** Preferred Reporting Items for Systematic Review and Meta-analysis (PRISMA) guideline.

**Additional file 2.** Search strategy.

**Additional file 3.** JBI critical appraisal checklist.

## Data Availability

All data generated and analyzed in the analysis process have been included in this manuscript.

## Abbreviations

AIDS: Acquired immune deficiency syndrome
AOR: Adjusted odds ratio
ART: Antiretroviral therapy
HAART: Highly active antiretroviral therapy
Hgb: Hemoglobin
HIV: Human immunodeficiency virus
JBI: Joanna Briggs Institute
PRISMA: Preferred Reporting Items for Systematic Review and Meta-analysis
SNNPRS: South Nation and Nationality Peoples Regional State
SSA: Sub-Saharan Africa
WHO: World Health Organization
