# Supplementary material for: Mortality and its association with CD4 cell count and hemoglobin level among children on antiretroviral therapy in Ethiopia: a systematic review and meta-analysis
Source: Trop Med Health. 2020 Sep 21;48:80. doi: 10.1186/s41182-020-00267-y (PMC7504851; doi:10.1186/s41182-020-00267-y)
Supplement: Supplementary file 2 — Additional file 2. Search strategy. [file 41182_2020_267_MOESM2_ESM.docx]

((mortality OR death OR survival OR treatment outcome OR attrition) AND (HIV/AIDS OR Human immune deficiency virus OR acquired immune deficiency syndrome OR ART OR antiretroviral therapy OR HAART OR highly active antiretroviral therapy) AND (prevalence OR proportion) AND (associated factors OR predictors OR determinants OR Hgb OR hemoglobin OR CD4 count) AND (child OR children OR pediatrics OR paediatrics OR infant OR neonate) AND (Ethiopia))
