# Supplementary material for: Mortality and its association with CD4 cell count and hemoglobin level among children on antiretroviral therapy in Ethiopia: a systematic review and meta-analysis
Source: Trop Med Health. 2020 Sep 21;48:80. doi: 10.1186/s41182-020-00267-y (PMC7504851; doi:10.1186/s41182-020-00267-y)
Supplement: Supplementary file 3 — Additional file 3. JBI critical appraisal checklist. [file 41182_2020_267_MOESM3_ESM.docx]

| Articles | **Critical appraisal criteria for cohort studies** | | | | | | | | | | | | | | | | | | | | | | | | Total score |
| --- | --- | --- | --- | --- | --- | --- | --- | --- | --- | --- | --- | --- | --- | --- | --- | --- | --- | --- | --- | --- | --- | --- | --- | --- | --- |
|  | Were the two groups similar and recruited from the same population | | | Were the exposures measured similarly? | | | Were confounding factors identified? | | | Were strategies to deal with confounding factors stated? | | | Were the groups/participants free of the outcome at the start of the study | | | Were the outcomes measured in a valid and reliable way? | | | Was follow up complete | | | Was appropriate statistical analysis used? | | |  |
|  | yes | No | NA | Yes | No | NA | Yes | No | NA | Yes | No | NA | Yes | No | NA | Yes | No | NA | Yes | No | NA | Yes | No | NA |  |
| Koye D et al. |  |  | √ | √ |  |  |  | √ |  | √ |  |  | √ |  |  | √ |  |  | √ |  |  | √ |  |  | 75% |
| Gebremedhin A et al. |  |  | √ | √ |  |  |  | √ |  | √ |  |  | √ |  |  | √ |  |  | √ |  |  | √ |  |  | 75% |
| Taye B. et al. |  |  | √ | √ |  |  |  | √ |  | √ |  |  | √ |  |  | √ |  |  | √ |  |  | √ |  |  | 75% |
| Asfawesen et al. |  |  | √ | √ |  |  |  | √ |  | √ |  |  | √ |  |  | √ |  |  | √ |  |  | √ |  |  | 75% |
| Dube et al. |  |  | √ | √ |  |  |  | √ |  | √ |  |  | √ |  |  | √ |  |  | √ |  |  | √ |  |  | 75% |
| Ebissa G. et al. |  |  | √ | √ |  |  |  | √ |  | √ |  |  | √ |  |  | √ |  |  | √ |  |  | √ |  |  | 75% |
| Sidamo NB et al. |  |  | √ | √ |  |  |  | √ |  | √ |  |  | √ |  |  | √ |  |  | √ |  |  | √ |  |  | 75% |
| Biru M et al. |  | × |  | √ |  |  |  | √ |  | √ |  |  | √ |  |  | √ |  |  | √ |  |  | √ |  |  | 62.5% |
| Atnafu H. and Wencheko E. |  |  | √ | √ |  |  |  | √ |  | √ |  |  | √ |  |  | √ |  |  | √ |  |  | √ |  |  | 75% |
| Netsanet W. et al. |  |  | √ | √ |  |  |  | √ |  | √ |  |  | √ |  |  | √ |  |  | √ |  |  | √ |  |  | 75% |
| Andargie A et al. |  |  | √ | √ |  |  |  | √ |  | √ |  |  | √ |  |  | √ |  |  | √ |  |  | √ |  |  | 75% |
| Edessa D. |  |  | √ | √ |  |  |  | √ |  | √ |  |  | √ |  |  | √ |  |  | √ |  |  | √ |  |  | 75% |
| Kedir AA. |  |  | √ | √ |  |  |  | √ |  | √ |  |  | √ |  |  | √ |  |  | √ |  |  | √ |  |  | 75% |
| Mokgatle MM et al. |  |  | √ | √ |  |  |  | √ |  | √ |  |  | √ |  |  | √ |  |  | √ |  |  | √ |  |  | 75% |
| Alebel A. et al |  |  | √ | √ |  |  |  | √ |  | √ |  |  | √ |  |  | √ |  |  | √ |  |  | √ |  |  | 75% |
| Arage G et al. |  |  | √ | √ |  |  |  | √ |  | √ |  |  | √ |  |  | √ |  |  | √ |  |  | √ |  |  | 75% |
|  |  | | |  | | |  | | |  | | |  | | |  | | |  | | |  | | |  |
