# Supplementary material for: Mortality and its association with CD4 cell count and hemoglobin level among children on antiretroviral therapy in Ethiopia: a systematic review and meta-analysis
Source: Trop Med Health. 2020 Sep 21;48:80. doi: 10.1186/s41182-020-00267-y (PMC7504851; doi:10.1186/s41182-020-00267-y)
Supplement: Supplementary file 3 [file 41182_2020_267_MOESM1_ESM.docx]

Section/topic	#	Checklist item
**TITLE**		
Title	1	Identify the report as a systematic review, meta-analysis, or both.
**ABSTRACT**		
Structured summary	2	Provide a structured summary including, as applicable: background; objectives; dat participants, and interventions; study appraisal and synthesis methods; results; limit implications of key findings; systematic review registration number.
**INTRODUCTION**		
Rationale	3	Describe the rationale for the review in the context of what is already known.
Objectives	4	Provide an explicit statement of questions being addressed with reference to partici outcomes, and study design (PICOS).
**METHODS**		
Protocol and registration	5	Indicate if a review protocol exists, if and where it can be accessed (e.g., Web addr registration information including registration number.
Eligibility criteria	6	Specify study characteristics (e.g., PICOS, length of follow-up) and report character language, publication status) used as criteria for eligibility, giving rationale.
Information sources	7	Describe all information sources (e.g., databases with dates of coverage, contact w additional studies) in the search and date last searched.
Search	8	Present full electronic search strategy for at least one database, including any limits repeated.
Study selection	9	State the process for selecting studies (i.e., screening, eligibility, included in system included in the meta-analysis).
Data collection process	10	Describe method of data extraction from reports (e.g., piloted forms, independently, for obtaining and confirming data from investigators.
Data items	11	List and define all variables for which data were sought (e.g., PICOS, funding sourc simplifications made.
Risk of bias in individual studies	12	Describe methods used for assessing risk of bias of individual studies (including spe done at the study or outcome level), and how this information is to be used in any d
Summary measures	13	State the principal summary measures (e.g., risk ratio, difference in means).
Synthesis of results	14	Describe the methods of handling data and combining results of studies, if done, inc (e.g., I^2) for each meta-analysis.
